# Evaluation of the Efficacy of Active Hexose Correlated Compound as an Adjuvant in Reducing Recurrence After Condyloma Cauterization

**DOI:** 10.3390/medicina61040622

**Published:** 2025-03-28

**Authors:** Ufuk Atlihan, Onur Yavuz, Can Ata, Huseyin Aytug Avsar, Tevfik Berk Bildaci, Ali Cenk Ozay, Burak Ersak, Ulas Solmaz, Selcuk Erkilinc

**Affiliations:** 1 Manisa Merkezefendi State Hospital, Manisa 45010, Turkey; 2 Faculty of Medicine, Dokuz Eylul University, Izmir 35020, Turkey; o-yavuz@hotmail.com; 3 Buca Seyfi Demirsoy Training and Research Hospital, Izmir 35020, Turkey; drcanata@yahoo.com; 4 Faculty of Medicine, Tınaztepe Galen University, Izmir 35020, Turkey; aytugavsar@hotmail.com; 5 Department of Gynecology, Faculty of Medicine, Izmir Democracy University, Izmir 35020, Turkey; berkbildaci@gmail.com; 6 Department of Obstetrics and Gynaecology, Cyprus International University, Nicosia 1010, Cyprus; dr.alicenk@gmail.com; 7 Faculty of Medicine, Ankara Bilkent City Hospital, University of Health Sciences, Ankara 06800, Turkey; burakersak@gmail.com; 8 Private Clinic, Izmir 35020, Turkey; drulassolmaz@gmail.com; 9 Department of Oncology, Faculty of Medicine, Izmir Democracy University, Izmir 35020, Turkey; selcukerkilinc@hotmail.com

**Keywords:** active hexose correlated compound, condylomata acuminate, human papillomavirus, relapse

## Abstract

*Background and Objectives*: Human papillomavirus (HPV) is one of the most prevalent sexually transmitted illnesses. HPV is responsible for genital condyloma lesions. A durable and effective systemic treatment regimen has not been established for HPV-related infections. In the present study, our purpose was to evaluate the role of active hexose correlated compound (AHCC) in preventing relapse in patients who underwent cauterization for condyloma accuminata. *Materials and Methods*: A total of 244 individuals admitted to our hospital between January 2019 and June 2022 were diagnosed as having condyloma acuminata, and those who underwent condyloma cauterization were evaluated retrospectively. We included 133 individuals who met the criteria. Patients who received AHCC were scheduled for follow-up examinations at regular intervals every three months. Patients were divided into two groups and analyzed based on whether they did or did not use AHCC. *Results*: The average age of AHCC non-users was significantly greater than that of AHCC users (*p* < 0.01). The number of condylomas and the maximum condyloma diameter of AHCC users before treatment were found to be significantly higher than in AHCC non-users (*p* = 0.006 and *p* = 0.004, respectively). Among participants with recurrence, the number and diameter of condylomas in AHCC users were significantly lower than in AHCC non-users (*p* = 0.019 and *p* = 0.042, respectively). *Conclusions*: Although the usage of AHCC is not expected to help prevent recurrence after the cauterization of condylomata acuminate in all patients, physicians may consider AHCC as a nutritional supplement and supportive therapy in the absence of other systemic treatments. Consequently, the duration of AHCC support necessary to optimize the effect of AHCC use on relapse prevention requires further evaluation on the basis of both target IFN-β levels and HPV infection status.

## 1. Introduction

The human papillomavirus (HPV) is regarded as one of the most prevalent sexually transmitted diseases (STDs) [1]. HPV infects the skin’s basal cells by penetrating the vaginal area’s microcracks in the skin. It causes epithelial differentiation, which leads to the formation of condyloma [2]. Ninety percent of lesions from genital condyloma are caused by HPV strains 6 and 11 [3]. Although they can be asymptomatic, they can also present clinical findings in the form of resistant recurrent infections [3,4]. There is a wide range of common clinical symptoms of HPV infections (e.g., vaginal discharge, burning, and bleeding in the anogenital area, or the appearance of condylomas in the genital area) [3,4]. The two peak periods for HPV-related infections are between the ages of 20 and 30 and between 40 and 50. During these periods, the incidence of HPV can increase by up to 80% [5]. The prevalence of HPV has been reported to be higher in people who have multiple sexual partners, a history of sexual intercourse at an early age, and immune system disorders [6,7,8]. Cryotherapy and cauterization are employed as the main modalities in the treatment of condyloma accuminata caused by HPV infection [6,7,8]. Although HPV vaccination provides almost total protection from HPV protection, its protective effect is reduced in patients who were already infected with HPV [9]. No durable and effective systemic treatment regimen has been established for high-risk HPV infections [9]. Created in Japan in 1992, active hexose correlated compound (AHCC) is a standardized and proprietary extract of grown Lentula edodes mycelia made up of α-glucan components (AHCC^®^, Amino Up, Ltd., Sapporo, Japan) [10,11,12,13,14,15]. Many human and animal investigations in the literature report various therapeutic effects of AHCC (e.g., preventing mechanisms that cause bacterial and viral infections, an antioxidant effect, anticancer activity, and the ability to modulate the immune system) [10,11,12,13,14,15]. Clinical studies report the effectiveness of AHCC in reducing infection risks and improving symptoms of current diseases [10,11,12,13,14,15]. However, there are no studies investigating the effect of AHCC as an adjuvant therapy on the recurrence of genital warts after treatment. In light of data from previous studies, the aim of the current research was to look at the use of AHCC from a different perspective by evaluating the role of AHCC in preventing relapse in patients who underwent cauterization for condyloma accuminata.

## 2. Materials and Methods

This investigation has a retrospective cross-sectional design. Every patient provided signed informed consent. The Declaration of Helsinki’s guiding principles were followed in the composition of the study, and the local ethics committee gave their approval (number: 2023/30-08). A total of 244 patients who were admitted to our hospital between January 2019 and June 2023, diagnosed as having condyloma accuminata and having undergone condyloma cauterization, were evaluated retrospectively. Patients who were immunocompromised, pregnant, had sexually transmitted infections other than HPV, had been administered an HPV vaccination in the past, had received an HPV vaccination after cauterization, had PAP smear test abnormalities, and whose PAP smear or HPV–PCR test data were not available were not included in the research; 111 patients were not included according to these criteria [suppressed immune systems (*n* = 6), pregnancy (*n* = 8), sexually transmitted infections other than HPV (*n* = 8), HPV vaccination in the past (*n* = 7), HPV vaccination after cauterization (*n* = 5), PAP smear test abnormalities (*n* = 15), PAP smear test results (*n* = 10), and 52 patients without HPV–PCR test data (*n* = 52)]. In total, the data of 133 individuals were scanned from the database and files. Data on potential risk factors, such as marital status, alcohol usage, age, condom usage, polygamy, smoking, and age at first sexual intercourse, were recorded. Before each yearly interview, regular smoking was defined as consuming 300 cigarettes in a year and at least 7 cigarettes daily on average over the previous week [16]. Regular alcohol use was defined as using alcohol >3 days a week, according to data from the United States National Institute on Alcohol Abuse and Alcoholism [17]. Patients who used AHCC (*n* = 47, 35.3%) and those who did not use AHCC (*n* = 86, 64.7%) were divided into two groups and analyzed. Patients who were using AHCC used 3 g/day oral AHCC as a supportive treatment for 6 months after condyloma cauterization. Patients who received AHCC were scheduled for follow-up examinations at regular intervals. They were called in for follow-up visits every 3 months for a period of 1 year. During the genital examination, we focused on the presence of new lesions, the size and number of existing lesions, and any signs of inflammation or irritation. Clear criteria for determining the healing of genital warts included the complete absence of visible lesions and normal epithelialization of the affected area. Recurrence was defined by the appearance of new warts or the reappearance of previously treated warts. We also monitored patients for early relapses, described as a recurrence that happens six months after starting therapy. Any instances of relapse within this period were carefully documented, noting the time of recurrence, the number and size of new lesions, and the overall condition of the genital area. The size of the warts was measured with a precision ruler at their widest point. This comprehensive follow-up approach ensured that we could accurately assess the effectiveness of AHCC in preventing the recurrence of genital warts. Statistical analysis was performed by employing the SPSS 26.0 software package (IBM Inc., Chicago, IL, USA). The normality of data distribution was evaluated using the Kolmogorov–Smirnov test. Non-normally distributed parameters were analyzed using the Mann–Whitney U test. The Chi-square test and Fisher’s exact test were used in the analysis of categorical data. The paired *t*-test was employed in the determination of changes before and after the treatment. The quantitative data of all patients are given as the mean ± standard deviation (SD) (minimum–maximum). The median value (maximum–minimum) is displayed for non-normally distributed values. Qualitative data are presented as numbers and percentages (%). To determine the link between two or more quantitative variables, regression analysis was used. Based on a 95% confidence interval (CI), the results were assessed. The threshold for statistical significance was set at *p* < 0.05.

## 3. Results

The average age of the participants was 34.6 ± 8 years, the mean age of first sexual intercourse was 23.8 ± 2.2 years, and the average number of partners in the last three years was 1.6 ± 0.9. The mean age of non-users of AHCC was significantly greater than that of AHCC users (*p* < 0.01). There was no significant difference between AHCC users and non-users in terms of age at first sexual intercourse, smoking, marital status, alcohol use, and number of partners in the last three years (Table 1).

Recurrence occurred in 66 (49.6%) patients. The number of condylomas and the maximum condyloma diameter of AHCC users before treatment were found to be significantly higher than those for AHCC non-users (*p* = 0.006 and *p* = 0.004, respectively). No significant differences were seen among participants who were AHCC users and non-users in terms of the presence of relapse, duration of relapse, and HPV types. In patients with recurrence, the number of condylomas and the condyloma diameters in AHCC users were significantly lower than those for AHCC non-users (*p* = 0.019 and *p* = 0.042, respectively) (Table 2).

A significant relationship was found between the number of condylomas before treatment, the diameter of condylomas before treatment, and the presence of recurrence (*p* = 0.001 and *p* = 0.001, respectively). No relationship was observed between the presence of HPV, smoking, condom use, age at first intercourse, and the presence of recurrence (Table 3).

## 4. Discussion

HPV, the most typical disease contracted during intercourse and the main virus linked with cancer, is considered among the world’s greatest threats to public health. Screening tests, vaccines, and early treatment for early diagnosis come to the fore in preventing HPV-related diseases. One of the most common HPV-related diseases is the presence of condyloma accuminata in the genital area. The results obtained in our study on the effectiveness of adjuvant AHCC, which is used to prevent the recurrence of condyloma accuminata due to HPV, are remarkable. In our study, although the condyloma diameter and number of condylomas before condyloma cauterization were higher in AHCC users, among the patients with recurrence the number of condylomas and condyloma diameters in AHCC users were found to be lower than those in AHCC non-users; however, no significant difference was detected between AHCC users and non-users in terms of the presence of recurrence. There are currently limited effective options in combating HPV-related infections. In young participants with strong immune systems, high-risk HPV infections will largely resolve without the need for any intervention within 6–24 months [18,19]. Ten percent or so of patients continue to have HPV infections [18,19]. The majority of choices include invasive surgeries or topical therapies that do not prevent HPV from returning to this location or other parts of the body that might be infected (such as the anus) [20]. There is no proven systemic modality with which to eliminate persistent HPV infections; however, there are 42 different natural and synthetic chemical compounds in the literature that may help eliminate HPV infections and related symptoms at an early stage, and prevent progression to aggressive cervical cancer [20]. Of these, 10 are synthetic and 32 are known as natural compounds [20]. When all participants were analyzed in this research, no significant difference was seen in terms of the presence of recurrence in AHCC users compared with that of non-users; however, HPV clearance was not directly evaluated in our study and, instead, condyloma acuminate relapses were evaluated as an indirect outcome. The results of two pilot studies involving healthy females who had been HPV-positive for more than two years confirm the conclusions of previous clinical studies; that supplementing with AHCC may help the host immune system fight off high-risk HPV infections [21]. A phase II randomized controlled trial supporting these two studies reported that, by modifying the human immune system, AHCC supplementation would eradicate high-risk HPV infections [22]. In our study, a significant decrease in the numbers and diameters of condylomas was found in AHCC users compared with those of non-users. Several therapeutic vaccines against HPV are currently in clinical trials. Additionally, clinical trials are testing different medications and formulations aimed at preventing the development of invasive illnesses linked to HPV infection [23]. After viral DNA is incorporated into the host cell genome, tumorigenic transformation occurs only after an apparent year or two of continuous HR-HPV infection. HPV infection is usually in an unintegrated episomal form during precancerous phases, which is advantageous since HPV may be easily eliminated with the use of the right medications and formulations. A number of these medications are undergoing clinical testing, and the results look good. An investigation was conducted on AHCC^®^, which aims to eradicate chronic HR-HPV infections, in a phase II randomized double-blind placebo-controlled crossover experiment. The study’s early findings demonstrated that IFN-β was correlated with the clearance of persistent HPV infections and that AHCC^®^ supplementation for at least six months was linked to a 60% effective removal of HPV infections. Treatment for HPV-related conditions such as vaginal intraepithelial neoplasia, anal intraepithelial neoplasia, and vulvar intraepithelial neoplasia involves the use of imiquimod [24]. It is presently undergoing a phase III study and is being evaluated as a potential substitute for surgical intervention in the treatment of CIN lesions. A randomized, placebo-controlled, and double-blind trial is being conducted on 2LPAPI^®^, an immune regulator, to assess its impact on the resolution of genital HR-HPV infections. By treating HPV infections and low-grade CIN lesions, dietary supplements including folic acid and polyphenol E (green tea extract) have successfully completed phase II studies, with the goal of preventing the development of cervical cancer in HPV-positive individuals. Studies are being carried out to ascertain the safety, effectiveness, and feasibility of curcumin in individuals with CIN3 lesions and to assess them for the presence of HR-HPV. Curcumin is another dietary supplement that is now in phase 0 [20]. Recently, certain therapeutic DNA vaccines have shown encouraging outcomes in the elimination of HPV. DNA vaccines are an innovative, attractive, and potentially useful antigen-specific immunotherapy technique. Compared to RNA or protein vaccines, they are safer, more stable, and easier to make. They can also sustain antigen expression in cells for a longer period of time. DNA vaccination is the process of injecting plasmid DNA encoding the target antigen (in this case, HPV E6 and E7) into host cells. Unlike live vector vaccinations, DNA vaccines do not cause the body to produce neutralizing antibodies for the injected plasmid, allowing them to be given repeatedly [25]. Human CRT coupled to HPV 16 E6, E7, and L2 (hCRTE6E7L2) served as the basis for the HPV DNA vaccine, which produced L2-specific neutralizing antibody responses as well as strong CD8+ T cell immunological responses specific to E6/E7 [25]. A recent investigation on a therapeutic vaccine including L2 and E7 found that the immunization may maintain a low T cell-mediated immune response while eliciting strong L2-specific IgG humoral responses [26]. In individuals with CIN2/3 lesions, the vaccines VGX-3100 and GX-188E efficiently target HPV 16 and 18 [27,28]. In individuals with CIN3 lesions, GX-188E specifically targets HPV antigens and elicits a strong E6/E7-specific IFN-producing T cell response [27]; VGX-3100 is the first therapeutic vaccine to show effectiveness against CIN2/3 caused by HPV 16 and HPV 18 [28]. As a result, these techniques could provide a non-surgical substitute and be useful in the management of HPV and related condyloma acuminata. In this current research, no relationship was detected between HPV types and the presence of condyloma relapse. In the paper by Kim et al., the relapse rate in high-risk HPV infections was 44.1%, similar to our results [29]. However, HPV positivity was determined as the relapse criterion in their study, whereas the presence of condyloma was the relapse criterion in our study. In our study, no relationships were detected between smoking and the presence of relapses. The research of Zhan et al. similarly reported that smoking was not a clinical factor associated with the presence of relapse [30]. Publications reporting that condom use reduces HPV transmission from people who are carriers of condyloma accuminata are contradictory [31]. In this current research, it could not be concluded that condom use was effective in preventing condyloma accuminata relapse. The reason for this might be that although condom use protects against sexually transmitted infections communicated through semen, urethral, vaginal, or cervical secretions, it does not protect against diseases transmitted through skin-to-skin contact (e.g., warts) [32]. No relationship was detected between alcohol use and condyloma acuminate relapse in our study. In a previous study evaluating this relationship, contrary to the present study’s results, HPV incidence infections were found to be high in patients with alcohol use [33]. In our study, only the presence of alcohol use was questioned as the reason for this difference. The frequency and duration of alcohol use of the patients were not categorized, similar to the study in the literature. Contrary to the data of two separate pilot studies and phase II studies [21,22], no significant conclusion was reached in the present study regarding the role of AHCC use in preventing relapse, and future research is needed to target IFN-β levels and optimize the duration of AHCC supplementation based on the individual’s HPV infection status. Optimizing and personalizing the duration of AHCC supplementation might help us determine the guiding treatment dose to prevent relapses in patients with condyloma accuminata. The fact that possible immune modulation mechanisms that might have supported the clearance of persistent HPV infections were not examined in the present study may be viewed as a restriction on our research. This is because the present study was retrospective, and hypotheses about the benefits of AHCC also depend on the modulation of host immune function. The strength of the present study was that previous studies focused on HPV clearance, whereas ours evaluated the effects of AHCC on preventing the relapse of condyloma acuminate.

## 5. Conclusions

Although the use of AHCC is not expected to help all patients prevent relapses after the cauterization of condyloma acuminate, physicians may consider AHCC as a nutritional supplement and supportive therapy in case more systemic therapies are not available. In conclusion, the duration of AHCC support necessary to optimize the effect of AHCC use on relapse prevention requires further evaluation based on both HPV infection status and target IFN-β levels.

## Figures and Tables

**Table 1:** Demographic features of patients.

Variables	AHCC Users *n* = 47 (35.3%)	AHCC Non-Users *n* = 86 (64.7%)	*p*
Age (years)	31 (22–48)	36.3 (24–63)	<0.01
Smoking	23/47 (48.9%)	47/86 (54.7%)	0.41
Alcohol use	25/47 (53.2%)	32/86 (37.2%)	0.09
Condom use	18/47 (38.2%)	31/86 (36%)	0.64
Age at first sexual intercourse (years)	23 (21–30)	24 (19–30)	0.6
Number of partners in the last 3 years	1 (0–4)	2 (0–5)	0.06
Marital Status Married Single	19 (40.4%) 28 (59.6%)	37 (43%) 49 (57%)	0.8

AHCC: active hexose correlated compound.

**Table 2:** Comparison of clinical findings of the groups.

Variables	AHCC User *n* = 47 (35.3%)	AHCC Non-User *n* = 86 (64.7%)	*p*
Number of condylomas before treatment	5 (2–10)	3 (1–9)	0.006
Maximum condyloma diameter before treatment (mm)	4 (2–12)	3 (1–7)	0.004
Presence of relapse	19 (40.4%)	47 (54%)	0.117
Relapse duration (months)	0 (0–8)	3 (0–12)	0.269
HPV Type HPV 16 and/or 18 HPV other	20 (42.6%) 27 (57.4%)	41 (47.7%) 45 (52.3%)	0.371
Number of condylomas in patients with recurrence	1 (0–5)	2 (0–7)	0.019
Condyloma diameter in patients with recurrence (mm)	1 (0–4)	2 (0–8)	0.042

AHCC: active hexose correlated compound, HPV: human papillomavirus.

**Table 3:** Risk factors for recurrent condyloma.

	**Median (Min–Max)**	**HR (95% CI)**	***p***
Number of condylomas before treatment	4 (1–10)	0.47 (0.32–0.68)	<0.001
Condyloma diameter before treatment (mm)	3 (1–12)	0.30 (0.17–0.54)	<0.001
HPV type HPV 16 and/or 18 HPV other	61 (45.8%) 72 (54.2%)	1.70 (0.58–4.96)	0.329
Smoking	70/133 (52.6%)	1.72 (0.60–4.92)	0.308
Latex condom use	49/133 (36.8%)	0.47 (0.15–1.44)	0.189
Age at first sexual intercourse (years)	24 (19–30)	1.19 (0.95–1.51)	0.125

HPV: human papillomavirus.

## Data Availability

The datasets used and/or analyzed during the current study are available from the corresponding author upon reasonable request, due to ethical committee restrictions.

## References

[1] Furumoto H., Irahara M. (2002). Human papilloma virus (HPV) and cervical cancer. J. Med. Investig..

[2] Doorbar J. (2006). Molecular biology of human papillomavirus infection and cervical cancer. Clin. Sci..

[3] Gunter J. (2003). Genital and perianal warts: New treatment opportunities for human papillomavirus infection. Am. J. Obstet. Gynecol..

[4] Thompson A.B., Flowers L.C. (2020). Human papillomavirus (HPV). Sexually Transmitted Infections in Adolescence and Young Adulthood: A Practical Guide for Clinicians.

[5] Chan P.K., Chang A.R., Yu M.Y., Li W.H., Chan M.Y., Yeung A.C., Cheung T.H., Yau T.N., Wong S.M., Yau C.W. (2010). Age distribution of human papillomavirus infection and cervical neoplasia reflects caveats of cervical screening policies. Int. J. Cancer.

[6] Dursun P., Senger S.S., Arslan H., Kuşçu E., Ayhan A. (2009). Human papillomavirus (HPV) prevalence and types among Turkish women at a gynecology outpatient unit. BMC Infect. Dis..

[7] Atlihan U. (2022). Analysis of Risk Factors for Human Papilloma Virus (HPV): A Survey Study. Arch. Clin. Psychiatry.

[8] Ufuk A., Muzaffer A., Ahmet Gökhan S., Senai A. (2023). Theoretical and Clinical Approaches in the Light of Current Developments in Health 4, Cervical Cancer Screening and Abnormal Smear Management.

[9] Koutsky L.A., Ault K.A., Wheeler C.M., Brown D.R., Barr E., Alvarez F.B., Chiacchierini L.M., Jansen K.U., Proof of Principle Study Investigators (2002). A controlled trial of a human papillomavirus type 16 vaccine. N. Engl. J. Med..

[10] Uno K., Kosuna K., Sun B., Fujii H., Wakame K., Chikumaru S., Ueda Y. (2000). Active Hexose Correlated Compound (AHCC) Improves Immunological Parameters and Performance Status of Patients With Solid Tumors. Biotherapy.

[11] Gao Y., Zhang D., Sun B., Fujii H., Kosuna K., Yin Z. (2006). Active hexose correlated compound enhances tumor surveillance through regulating both innate and adaptive immune responses. Cancer Immunol. Immunother..

[12] Takehito M., Kitadate K., Nishioka H., Wakame K. (2010). Basic and clinical studies on active hexose correlated compound. Biotechnology in Functional Foods and Nutraceuticals.

[13] Hirose A., Sato E., Fujii H., Sun B., Nishioka H., Aruoma O.I. (2007). The influence of active hexose correlated compound (AHCC) on cisplatin-evoked chemotherapeutic and side effects in tumor-bearing mice. Toxicol Appl. Pharmacol..

[14] Ishibashi H., Ikeda T., Tansho S., Ono Y., Yamazaki M., Sato A., Yamaoka K., Yamaguchi H., Abe S. (2000). Prophylactic efficacy of a basidiomycetes preparation AHCC against lethal opportunistic infections in mice. Yakugaku Zasshi J. Pharm. Soc. Jpn..

[15] Hunter R.J., Fujii H., Wakame K., Gaikwad A., Wolf J.K., Smith J.A. (2011). Evaluation of active hexose correlated compound (AHCC) in combination with pegylated liposomal doxorubicin for treatment of ovarian cancer. Int. J. Appl. Res. Nat. Prod..

[16] Gilliland F.D., Islam T., Berhane K., Gauderman W.J., McConnell R., Avol E., Peters J.M. (2006). Regular smoking and asthma incidence in adolescents. Am. J. Respir. Crit. Care Med..

[17] Chiva-Blanch G., Badimon L. (2019). Benefits and Risks of Moderate Alcohol Consumption on Cardiovascular Disease: Current Findings and Controversies. Nutrients.

[18] Huber J., Mueller A., Sailer M., Regidor P.A. (2021). Human papillomavirus persistence or clearance after infection in reproductive age. What is the status? Review of the literature and new data of a vaginal gel containing silicate dioxide, citric acid, and selenite. Womens Health.

[19] Shanmugasundaram S., Jianxin Y. (2017). Targeting persistent human papillomavirus infection. Viruses.

[20] Khamjan N.A., Beigh S., Algaissi A., Megha K., Lohani M., Darraj M., Kameli N., Madkhali F., Dar S.A. (2023). Natural and synthetic drugs and formulations for intravaginal HPV clearance. J. Infect. Public Health.

[21] Smith J.A., Mathew L., Gaikwad A., Rech B., Burney M.N., Faro J.P., Lucci J.A., Bai Y., Olsen R.J., Byrd T.T. (2019). From bench to bedside: Evaluation of AHCC supplementation to modulate the host immunity to clear high-risk human papillomavirus infections. Front. Oncol..

[22] Smith J.A., Gaikwad A.A., Mathew L., Rech B., Faro J.P., Lucci J.A., Bai Y., Olsen R.J., Byrd T.T. (2022). AHCC^®^ Supplementation to Support Immune Function to Clear Persistent Human Papillomavirus Infections. Front. Oncol..

[23] ClinicalTrials, a Resource Provided by the U.S. National Library of Medicine. https://clinicaltrials.gov/.

[24] Grimm C., Polterauer S., Natter C., Rahhal J., Hefler L., Tempfer C.B., Heinze G., Stary G., Reinthaller A., Speiser P. (2012). Treatment of cervical intraepithelial neoplasia with topical imiquimod: A randomized controlled trial. Obstet Gynecol..

[25] Peng S., Ferrall L., Gaillard S., Wang C., Chi W.Y., Huang C.H., Roden R.B.S., Wu T.C., Chang Y.N., Hung C.F. (2021). Development of DNA vaccine targeting E6 and E7 proteins of human papillomavirus 16 (HPV16) and HPV18 for immunotherapy in combination with recombinant vaccine boost and PD-1 antibody. mBio.

[26] Khallouf H., Grabowska A.K., Riemer A.B. (2014). Therapeutic vaccine strategies against human papillomavirus. Vaccines.

[27] Kim T.J., Jin H.T., Hur S.Y., Yang H.G., Seo Y.B., Hong S.R., Lee C.W., Kim S., Woo J.W., Park K.S. (2014). Clearance of persistent HPV infection and cervical lesion by therapeutic DNA vaccine in CIN3 patients. Nat. Commun..

[28] Trimble C.L., Morrow M.P., Kraynyak K.A., Shen X., Dallas M., Yan J., Edwards L., Parker R.L., Denny L., Giffear M. (2015). Safety, efficacy, and immunogenicity of VGX-3100, a therapeutic synthetic DNA vaccine targeting human papillomavirus 16 and 18 E6 and E7 proteins for cervical intraepithelial neoplasia 2/3: A randomised, double-blind, placebo-controlled phase 2b trial. Lancet.

[29] Kim J.K., Park Y.G., Kim B.G. (2022). Correlation between recurrence of anorectal condyloma acuminatum and human papilloma virus subtype. Genes Genom..

[30] Zhan M., Tong Z., Chen S., Miao Y., Yang Y. (2022). Establishing a prediction model for recurrence of condyloma acuminatum. Eur. J. Med. Res..

[31] Anic G.M., Lee J.H., Villa L.L., Lazcano-Ponce E., Gage C., José CSilva R., Baggio M.L., Quiterio M., Salmerón J., Papenfuss M.R. (2012). Risk factors for incident condyloma in a multinational cohort of men: The HIM study. J. Infect. Dis..

[32] Lyttle P.H., Thompson S.C. (2004). Maintaining sexual health in commercial sex workers in Australia: Condom effectiveness, screening, and management after acquiring sexually transmissible infections. Aust. N. Z. J. Public Health.

[33] Oh H.Y., Kim M.K., Seo S., Lee D.O., Chung Y.K., Lim M.C., Kim J., Lee C.W., Park S. (2015). Alcohol consumption and persistent infection of high-risk human papillomavirus. Epidemiol. Infect..

